# The Identity of the Meconium and Vernix Caseosa

**Published:** 1859-10

**Authors:** 


*The Identity of the Meconium and Vernix Caseosa.*—Prof. Forster says, "The general opinion respecting the meconium is, that it consists in a mixture of bile, intestinal mucus, and intestinal epithelium; but microscopical examination shows that besides the coloring-matter of the bile, it is composed chiefly of the vernix caseosa. For the most part it consists of small flat scales, which present all the characteristics of horny epithelial plates, completely corresponding to the horny scales of the vernix. Under the microscope, the meconium only differs from the vernix by the presence of the yellow coloring-matter, and the smaller number of fat globules. A proof of the identity is its containing minute hairs in just the same numbers as the vernix, which, indeed, without the microscope, may be separated from it by a needle. The horny scales could have no other source than the vernix, for the stomach and intestinal canal are lined with cylinder epithelium, and the mucous membrane of the mouth and œsophagus does not give rise to them. Besides these scales, we observe in the meconium, fatty globules of different sizes, crystals of cholesterine, and irregular yellow and brownish clodlets, which give the dark color to the meconium, and are doubtless biliary coloring-matter. The fatty globules are evidently of cutaneous sebaceous matter, and the cholesterine is in part derived from the bile, and in part from the decomposition of the vernix during its passage to and deposit in the rectum.

"The fœtus swallows from time to time some of the liquor amnii, having the vernix swimming in it, and the hairs and horny scales pass unchanged along the intestinal tract. Whether any of the sebaceous matter is taken up by the lacteals, may perhaps be determined by microscopical examination of the intestinal villi of the fœtus; and it would be interesting to determine, by numerous examinations of the intestinal canal, at what period this swallowing of the liquor amnii commences. As the elements of the vernix are only suspended in the liquor in small quantities, a large quantity of this must be gradually swallowed to lead to the amount of meconium usually present. The water must be soon absorbed from the stomach, as it is never found in it. The greater proportion is probably excreted by the kidneys, and again reaches the amnios. That it in nowise contributes material to the nourishment of the fœtus has been shown by Bischoff; but that does not prevent it serving some purpose in the economy. A regular examination of the entire contents of the intestinal canal in numerous fœtuses of different ages, is required to elucidate these points; and especially would such examination be of interest in the case of monsters. That the acephalæ have no meconium has long been known, and has usually been attributed to the absence of the liver. This would, however, only explain the absence of its dark color; and the meconium will only be wanting when, by reason of the malformation of the intestinal canal, the reception and transport of the liquor amnii holding the vernix caseosa are prevented.—*Wien Wochenschrift*, 1858, No. 32.—*Medical Times and Gazette.*

